# Changes in the incidence and outcome for early acute kidney injury in a cohort of Australian intensive care units

**DOI:** 10.1186/cc5949

**Published:** 2007-06-25

**Authors:** Sean M Bagshaw, Carol George, Rinaldo Bellomo, the ANZICS Database Management Committee

**Affiliations:** 1 Division of Critical Care Medicine, University of Alberta Hospital, Edmonton, Canada; 2 Department of Intensive Care, Austin Hospital, Melbourne, Australia; 3 Project Manager, ANZICS APD, Melbourne, Australia; 4 Department of Medicine, Melbourne University, Melbourne, Australia

## Abstract

**Introduction:**

There is limited information on whether the incidence of acute kidney injury (AKI) in critically ill patients has changed over time and there is controversy on whether its outcome has improved.

**Methods:**

We interrogated the Australian New Zealand Intensive Care Society Adult Patient Database to obtain data on all adult admissions to 20 Australian intensive care units (ICUs) for ≥ 24 hours from 1 January 1996 to 31 December 2005. Trends in incidence and mortality for ICU admissions associated with early AKI were assessed.

**Results:**

There were 91,254 patient admissions to the 20 study ICUs, with 4,754 cases of AKI, for an estimated crude cumulative incidence of 5.2% (95% confidence interval, 5.1 to 5.4). The incidence of AKI increased during the study period, with an estimated annual increment of 2.8% (95% confidence interval, 1.0 to 5.6, *P *= 0.04). The crude hospital mortality was significantly higher for patients with AKI than those without (42.7% versus 13.4%; odds ratio, 4.8; 95% confidence interval, 4.5 to 5.1; *P *< 0.0001). There was also a decrease in AKI crude mortality (annual percentage change, -3.4%; 95% confidence interval, -4.7 to -2.12; *P *< 0.001), however, which was not seen in patients without AKI. After covariate adjustment, AKI remained associated with a higher mortality (odds ratio, 1.23; 95% confidence interval, 1.14 to 1.32; *P *< 0.001) and there was a declining trend in the odds ratio for hospital mortality.

**Conclusion:**

Over the past decade, in a large cohort of critically ill patients admitted to 20 Australian ICUs, there has been a significant rise in the incidence of early AKI while the mortality associated with AKI has declined.

## Introduction

Acute kidney injury (AKI) is a common clinical problem in critically ill patients and typically portends an increase in morbidity and mortality [1]. Multiple epidemiologic investigations have provided a broad range of estimates of the incidence of AKI in critically ill patients [2-9]. Likewise, numerous studies have shown that AKI in the intensive care unit (ICU) is associated with high short-term and long-term case fatality rates, with dialysis dependence, with reduced quality of life and with excess utilization of health resources [2-6,9-20].

Regrettably, many of these studies suffer from limited generalizability as a result of disparities in the study methodology, the study population and the definitions of AKI. Moreover, no study has purposely evaluated or been capable of assessing trends in the incidence and outcome of AKI in critically ill patients over time, once changes in illness severity have been taken into account [21]. Accordingly, there is limited information on whether the incidence of AKI in the ICU has changed significantly over time and there is considerable controversy on whether its outcome has improved [22,23]. On the other hand, the Australian New Zealand Intensive Care Society (ANZICS) Adult Patient Database (APD) is a high-quality clinical database containing data from > 600,000 individual adult admissions to 135 ICUs from 1987 to the present that now captures approximately > 80% of all admissions to ICUs in Australia and New Zealand [24]. Twenty of these units have contributed data for a decade, making it possible to assess changes in incidence and outcome over a significant timespan.

We therefore interrogated the ANZICS APD to obtain information on the incidence and outcome of AKI in a cohort of critically ill patients from 20 Australian hospitals over a decade. We sought to describe the 10-year trend in the incidence of AKI at the time of or within 24 hours of admission to ICU, and the 10-year trend in the crude and adjusted hospital mortality rates associated with AKI.

## Methods

We conducted an observational surveillance cohort study to determine the incidence of and outcomes associated with AKI. We interrogated the ANZICS APD for all adult (age ≥ 18 years) ICU admissions for ≥ 24 hours with a diagnosis of AKI during the period from 1 January 1996 to 31 December 2005. In the event of multiple admissions for a particular patient, only the initial ICU admission was considered. Those patients readmitted to the ICU within 72 hours after their initial discharge were considered part of the index admission. We selected only those Australian ICUs that had continuously contributed data to the APD during this 10-year period. This cohort included 20 ICUs (nine tertiary referral centres, six metropolitan hospitals, four peripheral regional/rural hospitals and one private hospital).

### Identification of cases

We used two strategies to identify all ICU admissions associated with AKI. First, the APD has a prespecified data element for the presence of AKI [25]. For this data element, AKI was defined as an acute serum creatinine level ≥ 133 μmol/l or a 24-hour urine output < 410 ml and not having received prior renal replacement therapy. In addition, the APD verifies and validates any patient designated with AKI and a serum creatinine level < 200 μmol/l. Second, we evaluated the Acute Physiology and Chronic Health Evaluation (APACHE) III diagnostic codes for AKI in order to identify any additional patients. To further corroborate admissions with AKI, all identified patients were then referenced with APACHE II and APACHE III diagnostic codes for chronic renal replacement therapy and/or kidney transplant.

### Data collection

Standard demographic, clinical and physiologic data were retrieved. Demographic information included age, sex, dates of admission to the hospital and the ICU, and source of admission. Clinical data encompassed the primary diagnosis, surgical status, the presence of selected comorbid illnesses and a need for mechanical ventilation. Data on kidney function extracted included the peak serum creatinine and urea, and the total 24-hour urine output within the first 24 hours of ICU admission [25]. Severity of illness during the first 24 hours of ICU admission was assessed using the APACHE II, APACHE III and Simplified Acute Physiology Score II scoring systems [26,27].

Pre-existing comorbid illnesses were defined using the chronic health evaluation for the APACHE II, APACHE III and Simplified Acute Physiology Score II scoring systems, as outlined in the ANZICS APD data dictionary [25].

Several primary admission diagnostic categories were created [25]. Sepsis/septic shock encompassed admissions for primarily sepsis-related diagnoses, and included sepsis associated with pneumonia, gastrointestinal disease, urinary tract infections, central nervous system infections, soft tissue infections, and the ANZICS APD-specific diagnostic code additions for sepsis with shock of undetermined source. A primary cardiac diagnosis encompassed nonsurgical admissions with cardiogenic shock, cardiac arrest, congestive heart failure and acute myocardial infarction. A primary hepatic diagnosis included admission with hepatic failure or liver transplant. A diagnosis of gastrointestinal haemorrhage included bleeding due to peptic ulcers, diverticulosis and varices. A metabolic/poisoning diagnosis incorporated nonoperative causes of metabolic coma, diabetic ketoacidosis, drug overdoses or other endocrinopathies. A primary respiratory diagnosis encompassed primary respiratory arrests, aspiration syndrome, noncardiogenic pulmonary oedema, exacerbations of chronic obstructive pulmonary disease or asthma, and pulmonary embolism. A primary neurologic diagnosis incorporated stroke, intracerebral haemorrhage, subarachnoid haemorrhage, epidural haematoma or other neurologic cause for coma.

### Clinical outcomes

Outcomes extracted from the APD included an incidence of early AKI at or within 24 hours of ICU admission (as a proportion of all ICU admissions) and the hospital mortality rate. If patients were readmitted to the ICU prior to hospital discharge, subsequent ICU admissions were not included in the analysis of mortality. The ICU and hospital lengths of stay and the hospital discharge location were also evaluated.

### Statistical analysis

Analysis was performed using Stata version 8.2 (Stata Corp, College Station, TX, USA). In the event of missing data values, data were not replaced or estimated. Normally or near-normally distributed variables are reported as means with standard deviations and were compared by Student's *t *test. Non-normally distributed continuous data are reported as medians with interquartile ranges and were compared by the Mann–Whitney *U *test. Categorical data are reported as proportions and were compared using Fisher's exact test.

Incidence estimates for early AKI on admission to the ICU were calculated as a proportion of all admissions to the ICU with 95% confidence intervals (CIs). Incidence estimates are presented as cumulative over 10 years, as time-stratified by 2-year intervals and as stratified by demographics, baseline characteristics and primary diagnosis. To determine changes over time, parametric and nonparametric tests for trend were performed as appropriate.

The estimated annual percentage changes in the incidence of AKI were determined by fitting a straight-line regression of the natural logarithm of the rates, with the calendar year used as an independent variable. The estimated annual percentage change was equal to [100 × (exp(*b*) - 1)], where *b *represents the slope of the regression. If the estimated annual percentage change is statistically greater than zero, then the incidence rate has an increasing trend over the study period [28].

Multivariable logistic regression was used to calculate the adjusted odds ratios (ORs) with 95% CIs for the association of AKI at ICU admission with hospital mortality. The variables age, sex, comorbidity, surgical/medical admission, primary diagnosis, severity of illness (APACHE II score), mechanical ventilation and hospital site were included. Model fit was assessed by the goodness-of-fit test and discrimination was assessed by the area under the receiver operator characteristic curve. *P *< 0.05 was considered statistically significant for all comparisons.

## Results

During the 10-year study period, 91,254 patients were admitted to the 20 study ICUs. Overall, these patients had a median (interquartile range) age of 64.1 (49 to 74.1) years, 60.6% were male, 21.5% had evidence of comorbid disease, 50.4% were medical admissions and the initial mean (± standard deviation) APACHE II score was 16.4 (± 7.8).

### Incidence

**Figure 1:**
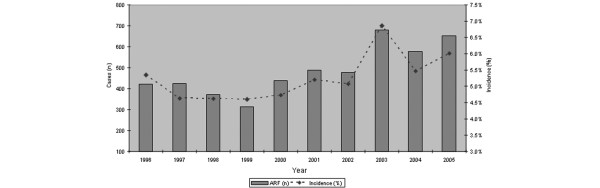
Summary of cases of acute kidney injury and incidence from the Australia New Zealand Intensive Care Society Adult Patient Database, 1996–2005. ARF, acute renal failure.

In total, 4,754 patients had a diagnosis of AKI at the time of or during the first 24 hours after ICU admission. This translated into an estimated crude cumulative incidence of 5.2% (95% CI, 5.1 to 5.4). The range in incidence was 4.6 to 6.9%. There was a significant increasing trend in incidence over the study period, with an estimated annual percentage increment of 2.8% (95% CI, 1.0 to 5.6; *P *= 0.04) (Figure 1). The incidence was significantly greater for admissions in 2001–2005 compared with admissions during 1996–2000 (5.6% versus 4.8%; OR, 1.16; 95% CI, 1.10 to 1.23; *P *< 0.0001); this difference persisted after taking into account the apparent high 6.9% incidence in 2003 (5.2% versus 4.8%; OR, 1.10; 95% CI, 1.03 to 1.16; *P *= 003).

### Demographics

Older patient age was associated with a higher incidence of AKI (Table 1). There were no significant changes in incidence of AKI stratified by age. There was, however, a nonsignificant increase in incidence for patients aged ≥ 75 years (annual percentage change, 2.0%; 95% CI, -0.5 to 4.6; *P *= 0.1). There was no significant difference in the cumulative incidence stratified by sex (5.1% for males versus 5.4% for females; OR, 0.96; 95% CI, 0.90 to 1.01; *P *= 0.12) or evidence for a change over the study period (Table 1).

**Table 1:** Incidence rates (95% confidence intervals) of acute kidney injury stratified by two-year intervals, age, sex and comorbid illness from the Australia New Zealand Intensive Care Society Adult Patient Database 1996–2005

Covariate	Cumulative		Incidence rates per two-year period				
	
	Cases (*n *= 4,754)	Incidence (5.2 (5.1–5.4))	1996/1997 (*n *= 849)	1998/1999 (*n *= 684)	2000/2001 (*n *= 926)	2002/2003 (*n *= 1,158)	2004/2005 (*n *= 1,137)
Age							
18–49 years	838	3.6 (3.3–3.8)	3.4 (2.8–3.9)	3.3 (2.8–3.9)	3.7 (3.2–4.3)	3.9 (3.4–4.4)	3.5 (3.0–3.9)
50–64 years	1,026	4.5 (4.3–4.8)	4.3 (3.7–4.9)	4.3 (3.6–4.9)	4.0 (3.5–4.6)	5.1 (4.4–5.7)	4.9 (4.3–5.4)
65–74 years	1,304	5.7 (5.4–6.0)	5.7 (5.0–6.3)	4.8 (4.2–5.5)	5.4 (4.7–6.0)	6.6 (5.9–7.3)	5.9 (5.2–6.5)
≥ 75 years	1,545	7.5 (7.2–7.9)	7.0 (6.1–7.9)	6.8 (5.9–7.7)	7.2 (6.4–8.0)	8.8 (8.0–9.6)	7.4 (6.7–8.1)
Sex							
Male	2,831	5.1 (4.9–5.3)	4.9 (4.5–5.4)	4.5 (4.0–4.9)	4.8 (4.4–5.2)	5.7 (5.3–6.1)	5.5 (5.1–5.9)
Female	1,923	5.4 (5.1–5.6)	5.0 (4.5–5.5)	4.9 (4.3–5.4)	5.2 (4.7–5.8)	6.4 (5.8–6.9)	5.1 (4.7–5.6)
Comorbid illness							
None	3,335	4.7 (4.5–4.8)	4.2 (3.9–4.6)	4.1 (3.7–4.4)	4.3 (4.0–4.7)	5.5 (5.1–5.9)	4.9 (4.5–5.1)
1 comorbidity	1,067	6.8 (6.4–7.2)	6.6 (5.7–7.4)	6.1(5.1–7.0)	6.8 (5.9–7.6)	7.6 (6.7–8.5)	6.9 (6.1–7.7)
2 comorbidities	312	8.7 (7.7–9.6)	12.2 (9.6–14.8)	7.6 (5.3–9.9)	8.6 (6.7–10.6)	7.5 (5.8–9.3)	7.9 (6.1–9.8)
≥ 3 comorbidities	40	12.4 (8.8–16.0)	12.5 (4.1–20.8)	16.4 (6.8–26.0)	15.0 (7.0–23.0)	9.2 (2.6–15.9)	7.3 (0–15.6)
Any comorbidity	1,419	7.2 (6.9–7.6)	7.6 (6.7–8.4)	6.5 (5.6–7.4)	7.3 (6.5–8.1)	7.6 (6.8–8.4)	7.1 (6.3–7.8)
Comorbid conditions							
Cardiovascular	553	6.3 (5.8–6.8)	7.7 (6.4–9.0)	5.0 (3.9–6.0)	6.3 (5.2–7.3)	5.7 (4.7–6.8)	6.7 (5.5–7.8)
Respiratory	404	6.9 (6.3–7.6)	6.2 (4.9–7.6)	5.4 (3.9–6.9)	8.0 (6.4–9.6)	8.3 (6.8–9.9)	6.2 (4.9–7.5)
Liver	230	12.1 (10.6–13.6)	12.1 (8.8–15.6)	12.8 (8.5–17.1)	12.1 (8.7–15.5)	13.2 (9.9–16.5)	10.9 (8.3–13.6)
Metastatic cancer	143	8.2 (6.9–9.5)	8.4 (5.3–11.6)	9.4 (5.8–13.1)	7.7 (4.6–10.9)	8.6 (5.9–11.3)	7.3 (4.9–9.6)
Haematologic malignancy	134	10.5 (8.8–12.2)	15.6 (10.5–20.7)	16.2 (10.3–22.1)	9.9 (6.3–13.6)	6.9 (3.9–9.9)	8.7 (5.9–11.6)
Immunocompromised	351	8.2 (7.4–9.0)	8.9 (7.0–10.8)	8.7 (6.6–10.8)	8.3 (6.5–10.1)	7.9 (6.2–9.6)	7.1 (5.4–8.9)

### Patient characteristics

The incidence of AKI was considerably higher when stratified by both the presence of pre-existing comorbid illness and by specific comorbid illnesses (Table 1). There was a nonsignificant trend for an increase in the incidence of AKI for patients with no comorbid illness (annual percentage change, 2.9%; 95% CI, -0.4 to 6.2; *P *= 0.08). There were no significant changes, however, in incidence stratified by the number of comorbid diseases. For the specific comorbid diseases evaluated, all were associated with a significantly higher incidence AKI. In particular, comorbid liver disease (OR, 2.58; 95% CI, 2.24 to 2.98; *P *< 0.0001) and haematologic malignancy (OR, 2.18; 95% CI, 1.82 to 2.61; *P *< 0.0001) showed the highest risk. During the study period, only haematologic malignancy showed a significant change in incidence of AKI, characterized by a decreasing trend (annual percentage change, -65%; 95% CI, -86 to -12; *P *= 0.03).

Nonelective admissions compared with elective admissions were associated with a higher incidence of AKI (7.2% versus 1.7%; OR, 4.6; 95% CI, 4.20 to 5.04; *P *< 0.0001) (Table 2). Over the study period, there was a nonsignificant but increasing trend in the incidence of AKI for elective ICU admissions (annual percentage change, 6.4%; 95% CI, -1.2 to 14.6; *P *= 0.09). There was no change for nonelective admissions, however.

**Table 2:** Incidence rates (95% confidence intervals) of acute kidney injury stratified by two-year intervals, and admission characteristics from the Australia New Zealand Intensive Care Society Adult Patient Database 1996–2005

Covariate	Cumulative		Incidence rates per two-year period				
	
	Cases (*n *= 4,754)	Incidence (5.2 (5.1–5.4))	1996/1997 (*n *= 849)	1998/1999 (*n *= 684)	2000/2001 (*n *= 926)	2002/2003 (*n *= 1,158)	2004/2005 (*n *= 1,137)
Admission category							
Elective	544	1.7 (1.5–1.8)	1.4 (1.1–1.7)	1.0 (0.7–1.3)	1.5 (1.3–1.8)	2.4 (2.1–2.8)	1.8 (1.4–2.1)
Nonelective	4,209	7.2 (7.0–7.4)	7.0 (6.5–7.4)	6.8 (6.3–7.3)	7.2 (6.7–7.7)	7.9 (7.5–8.4)	7.0 (6.6–7.5)
Admission type							
Surgical	957	2.1 (2.0–2.2)	2.5 (2.2–2.8)	2.0 (1.7–2.3)	1.7 (1.4–2.0)	2.6 (2.3–2.9)	1.9 (1.7–2.2)
Medical	3,752	8.3 (8.0–8.5)	7.5 (7.0–8.1)	7.6 (7.0–8.2)	8.5 (7.9–9.1)	9.3 (8.7–9.9)	8.1 (7.6–8.6)
Surgical subcategory							
Cardiovascular	376	1.6 (1.4–1.8)	1.9 (1.5–2.2)	1.8 (1.4–2.3)	1.3 (1.0–1.6)	1.7 (1.4–2.1)	1.3 (1.0–1.6)
Trauma	72	1.7 (1.3–2.1)	2.9 (1.6–4.2)	1.6 (0.7–2.4)	1.4 (0.7–2.1)	1.5 (0.7–2.2)	1.7 (0.8–2.6)
Diagnostic category							
Sepsis/septic shock	1,109	19.5 (18.5–20.5)	23.0 (20.0–26.0)	19.1 (16.0–22.2)	20.6 (18.1–23.1)	22.9 (20.6–25.2)	15.4 (13.8–17.0)
Cardiac	658	11.2 (10.4–12.0)	9.6 (8.0–11.3)	12.5 (10.3–14.6)	10.9 (9.0–12.7)	11.8 (10.0–13.6)	11.6 (9.9–13.3)
Hepatic	362	8.0 (7.2–8.8)	7.4 (5.6–9.2)	7.5 (5.5–9.5)	9.4 (7.3–11.4)	7.9 (6.1–9.6)	7.9 (6.5–9.4)
Gastrointestinal bleeding	108	6.1 (5.0–7.3)	4.7 (2.3–7.1)	5.3 (2.2–8.4)	5.7 (3.0–8.4)	8.9 (6.1–11.7)	5.5 (3.6–7.4)
Metabolic/poisoning	191	3.9 (3.4–4.4)	3.1 (1.9–4.2)	3.3 (2.1–4.6)	4.2 (2.9–5.5)	3.9 (2.7–5.1)	4.5 (3.4–5.7)
Respiratory	383	3.4 (3.0–3.7)	2.7 (2.1–3.4)	3.0 (2.3–3.8)	3.7 (3.0–4.5)	4.2 (3.4–5.0)	3.1 (2.4–3.8)
Neurologic	101	2.1 (1.7–2.5)	1.9 (1.0–2.8)	2.0 (1.0–3.1)	2.1 (1.2–3.0)	2.0 (1.2–2.9)	2.3 (1.4–3.1)

Medical admissions compared with primarily surgical admissions were associated with a higher incidence of AKI (8.3% versus 2.1%; OR, 4.11; 95% CI, 3.82 to 4.42; *P *< 0.0001) (Table 2). There was a nonsignificant decreasing trend in the incidence of AKI associated with cardiovascular surgery (annual percentage change, -4%; 95% CI, -8.9 to 12; *P *= 0.1) and a significant decrease in the incidence of AKI associated with trauma (annual percentage change, -8%; 95% CI, -13 to -2.3; *P *= 0.009) over the study period.

Several admission diagnoses were associated with an increased incidence of AKI (Table 2). There were no significant changes in incidence by diagnostic category over the study period, with the exception of an increasing trend in incidence of AKI associated with metabolic/poisoning diagnoses (annual percentage change, 5.5%; 95% CI, 0.6–10.7; *P *= 0.03).

Details of kidney function and severity of illness scores for the first 24 hours after ICU admission for patients with AKI are presented in Table 3.

**Table 3:** Summary of kidney function for patients admitted to the intensive care unit with acute kidney injury from the Australia New Zealand Intensive Care Society Adult Patient Database 1996–2005

Kidney function parameter	Overall	1996/1997	1998/1999	2000/2001	2002/2003	2004/2005
Incidence of acute kidney injury (%) (95% confidence interval)	5.2 (5.1–5.4)	5.0 (4.6–5.3)	4.6 (4.3–4.9)	5.0 (4.7–5.3)	6.0 (5.7–6.3)	5.3 (5.0–5.6)
APACHE II score (mean (standard deviation))	27 (8.4)	27.8 (8.5)	27.1 (8.4)	27.1 (8.4)	26.6 (8.6)	26.7 (8.0)
Simplified Acute Physiology Score II score (mean (standard deviation))	52.3 (18.6)	56 (18.8)	55.4 (18.7)	50.9 (18.1)	50.4 (18.9)	50.9 (17.8)
Serum creatinine (μmol/l) (median (interquartile range))	245 (170–362)	261 (200–390)	255 (190–365)	240 (148–356)	230 (157–353)	243 (170–360)
Serum creatinine ≥ 133 μmol/l (%)	86.8	93.3	91	78.4	84	89.1
Serum urea (mmol/l) (mean (standard deviation))	20.4 (12.5)	21.7 (13.1)	20.4 (11.6)	20.4 (12.3)	19.6 (12.5)	20.1 (12.5)
Urine output (ml/hour) (median (interquartile range))	40 (11.6–95)	23.6 (9.6–90)	22.9 (9–82)	50.6 (14.1–105)	51 (15–105)	36.8 (11.6–89)
Oliguria (< 410 ml/24 hour) (%)	38.7	48.7	47.4	32.4	32.3	37.1

APACHE, Acute Physiology and Chronic Health Evaluation.

### Mortality

**Figure 2:**
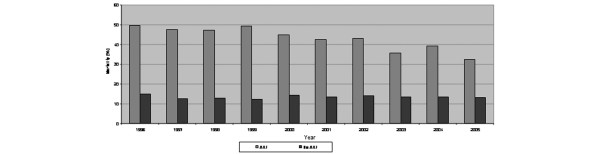
Summary of crude mortality for patients with and without acute kidney injury from the Australia New Zealand Intensive Care Society Adult Patient Database, 1996–2005. ARF, acute renal failure.

The crude hospital mortality was significantly higher for patients with AKI than those without (42.7% versus 13.4%; OR, 4.8; 95% CI, 4.5 to 5.1; *P *< 0.0001) (Table 4 and Figure 2). There was, however, a significant decrease over time in the crude mortality rate associated with AKI (annual percentage change, -3.4%; 95% CI, -4.7 to -2.12; *P *< 0.001). There was no change for those without AKI over the study period. The presence of AKI remained associated with higher mortality after adjustment for age, sex, comorbidity, surgical/medical admission, primary diagnosis, severity of illness (APACHE II score), mechanical ventilation and hospital site (OR, 1.39; 95% CI, 1.3 to 1.5; *P *< 0.001). Over the study period, there was a trend for decreasing ORs for death associated with AKI.

**Table 4:** Crude and age, sex, comorbidity and severity of illness-adjusted odds ratios (95% confidence intervals) for the association of acute kidney injury and hospital mortality stratified by two-year intervals from the Australia New Zealand Intensive Care Society Adult Patient Database 1996–2005

Mortality outcome	Overall	1996/1997	1998/1999	2000/2001	2002/2003	2004/2005
Crude	4.80 (4.5–5.1)	6.03 (5.2–7.0)	6.47 (5.5–7.6)	4.74 (4.1–5.4)	3.94 (3.5–4.5)	4.11 (3.6–4.7)
Age and sex adjusted	4.41 (4.1–4.7)	5.63 (4.9–6.5)	6.05 (5.2–7.1)	4.40 (3.8–5.1)	3.56 (3.1–4.0)	3.73 (3.3–4.2)
Age, sex and comorbidity adjusted	4.28 (4.2–4.6)	5.29 (4.6–6.1)	5.90 (5.0–6.9)	4.21 (3.7–4.8)	3.54 (3.1–4.0)	3.66 (3.2–4.2)
Age, sex, comorbidity and severity adjusted	1.42 (1.3–1.5)	1.47 (1.2–1.7)	1.48 (1.2–1.8)	1.59 (1.3–1.9)	1.25 (1.1–1.5)	1.39 (1.2–1.6)
Adjusted odds ratio^a^	1.39 (1.3–1.5)	1.54 (1.3–1.9)	1.64 (1.3–2.0)	1.38 (1.2–1.6)	1.20 (1.02–1.4)	1.33 (1.1–1.6)

^a^Adjustment for age, sex, comorbidity, surgical/medical admission, primary diagnosis, severity of illness (Acute Physiology and Chronic Health Evaluation II score), mechanical ventilation and hospital site. Goodness-of-fit test, *P *= 1.0; area under the receiver operator characteristic curve, 0.84.

### Additional clinical outcomes

Those patients with AKI had a longer median (interquartile range) stay in both the ICU and the hospital than those without AKI (Table 5). Specifically, AKI increased both the duration of the ICU stay (4.4 (2.1–9.5) days for AKI versus 2.6 (1.7 to 4.9) days for no AKI, *P *< 0.0001) and of the hospital stay (14.2 (6.5 to 28.9) days for AKI versus 11.7 (7.0 to 21.9) days for no AKI, *P *< 0.0001). The total duration of stay was also significantly longer in survivors to hospital discharge stratified by AKI than in nonsurvivors (19.8 (10.8 to 37.2) days versus 11.9 (7.2 to 21.9) days, *P *< 0.0001). There were no significant changes in ICU or hospital lengths of stay over the study period.

**Table 5:** Clinical outcomes in critically ill patients admitted with acute kidney injury from the Australia New Zealand Intensive Care Society Adult Patient Database 1996–2005

	Overall	1996/1997	1998/1999	2000/2001	2002/2003	2004/2005
Intensive care unit stay (days) (median (interquartile range))						
Dead	3.4 (1.8–8.5)	3.0 (1.6–8.2)	3.2 (1.6–8.6)	3.1 (1.8–7.9)	3.5 (1.8–8.4)	4.3 (1.9–9.8)
Alive	5.0 (2.7–10.0)	5.6 (2.9–11.8)	6.1 (2.8–11.7)	5.0 (2.6–10.1)	4.7 (2.4–9.0)	4.8 (2.8–9.5)
Hospital stay (days) (median (interquartile range))						
Dead	7.5 (2.9–17.7)	6.5 (2.5–15.7)	7.1 (2.9–16.5)	7.8 (3.0–18.8)	7.8 (2.9–19.7)	8.0 (3.2–18.6)
Alive	19.8 (10.8–37.2)	21.9 (11.8–40.1)	20.5 (11.1–35.4)	19.7 (11.0–37.6)	18.7 (10.0–35.2)	18.8 (9.9–37.6)
Discharge location of survivors (%)						
Home	74.9	75.5	70.9	74.9	76.1	75.1
Transfer to another acute care hospital	16.6	14.7	19.8	17.2	14.8	17.6
Rehabilitation/long-term care facility	8.6	9.9	9.3	7.8	9.1	7.3

The hospital discharge location was significantly different for patients with AKI compared with those patients with no AKI (Table 5). Fewer patients with AKI were discharged home than patients without AKI (74.8% versus 84.8%, *P *< 0.001); instead, AKI patients were more likely to have been transferred to another acute care hospital (16.6% versus 9.6%, *P *< 0.001) or a rehabilitation facility (8.6% versus 5.5%, *P *< 0.001). There were no significant changes in hospital discharge location over the study period.

## Discussion

We conducted a 10-year observational study of > 90,000 ICU admissions to 20 ICUs in Australia, using a high-quality clinical database, to evaluate trends in the incidence and mortality associated with AKI. We found that approximately 5.2% of critically ill patients are diagnosed with AKI at the time of ICU admission and that the incidence of AKI has increased significantly over the past decade. We also found that the incidence of AKI associated with admission for metabolic diagnoses and/or poisonings has increased but that the incidence has declined in those patients admitted with trauma or haematologic malignancies. We confirmed that the mortality rate associated with AKI remains high and that the increased risk of death associated with AKI persisted after adjustment for several relevant covariates. Finally, we found that, despite an increasing incidence, the multivariate adjusted odds of death associated with AKI have shown a declining trend over the 10-year study period.

Numerous epidemiologic investigations have estimated the occurrence and associated burden of AKI on clinical outcomes and health resources in critically ill patients [1-5,7,8,11,12,15,19,29]. Very few studies, however, have assessed whether the incidence or outcomes associated with AKI have changed over time [21,30,31]. Moreover, these studies are often limited to a single centre and compare two discrete periods in time separated by several years [21]. Two recent large epidemiologic investigations using administrative databases showed similar patterns of increasing incidence and decreasing mortality with AKI; however, these studies are limited by focusing on all hospitalized patients rather than on only those admitted to ICU. Overall, this paucity of data examining for trends in incidence over time is unfortunate when taking into account the poor clinical outcome and high cost of care for critically ill patients with AKI [10,13,32].

The key findings from our study, specifically that AKI is common and its occurrence is on the rise, may have important health resource and economic implications. For instance, our data support the findings of prior investigations showing that AKI may play a role in prolonging the duration of stay in the ICU and the hospital and may lead to higher rates of hospital discharge to long-term care or rehabilitation facilities [2,11]. One consequence of these differences in clinical outcomes would undoubtedly be the consumption of considerable health resources [10,13,33,34].

Additionally, there has been considerable controversy as to whether the clinical outcomes – in particular, mortality associated with AKI – have improved [22,23]. For example, Ympa and colleagues reported in a systematic review that mortality associated with AKI has shown no consistent change over several decades [23]. Regrettably, their study was highly prone to bias and was limited by only reporting crude mortality rates across those studies included and by the inability to show equivalent illness severity. On the contrary, we have found over the past decade that the mortality associated with AKI, when adjusted for covariates, has shown a declining trend. Whether this decline can be attributed to an improvement in the overall care of critically ill patients or by specific interventions or therapies aimed at those with AKI remains unknown [35-38]. This decline in mortality has, however, occurred despite reported changes to the clinical profile and characteristics of critically ill patients with AKI [8,22]. Observational studies suggest that critically ill patients with AKI are increasingly older, have more comorbid disease, are more probably septic, and have greater severity of illness and organ failure [2,6].

In our study, we evaluated for changes in the profile and characteristics of patients that might have also corresponded to changes in the incidence of AKI. We found no notable trends when stratified by age or the presence of comorbid illness, with the exception of a decline in AKI associated with haematologic malignancy. Similarly, while ICU admissions for sepsis, acute cardiac conditions and hepatic failure were all associated with a higher risk for AKI, there were no significant trends in incidence for these conditions over the study period, with the exception of a rise in AKI associated with admissions for acute metabolic/poisoning conditions. Interestingly, however, we found a declining trend in the incidence of AKI associated with trauma. While the number of cases of AKI associated with trauma in our study was relatively small, there are plausible explanations for this finding – such as an increase in regionalized trauma systems [39,40], advancements in prehospital care [41] and earlier identification of patients at high risk for AKI, due to conditions such as rhabdomyolysis, with initiation of timely prophylactic interventions [42,43].

There are both limitations and strengths to our study. First, the definition of AKI used in our study, as mandated by the APD, will invariably influence the overall incidence estimates. We have, however, used several measures to capture patients designated with acute reductions in kidney function consistent with the syndrome of AKI. Second, we were unable to determine the precise prevalence of chronic kidney disease with the exception of those patients requiring chronic renal replacement therapy. This may also influence the overall incidence estimates. To minimize misclassification, we have attempted to exclude all patients with known end-stage renal disease or all admissions to the ICU that were related to kidney transplantation. Reassuringly, our incidence estimates appear largely consistent with the current literature [1]. Third, we were unable to provide estimates of the proportion of patients requiring acute renal replacement therapy. Fourth, we were only able to collect data on patients within the first 24 hours of admission to the ICU. The incidence estimates of AKI therefore probably underestimate the true burden of AKI as some patients would have developed delayed AKI several days after admission [44]. Moreover, we are unable to assess long-term outcome or renal recovery. On the other hand, this is by far the largest study of AKI ever conducted in terms of the overall screened population and target cohort, and the only study where outcomes and illness severity could be studied in the same units over an entire decade.

## Conclusion

To our knowledge, we conducted the first large multicentre study of AKI in critically ill patients to evaluate long-term trends in incidence and mortality. In this heterogeneous cohort of critically ill patients, we found a significant rise in the incidence of AKI. Moreover, despite modest changes in the profile of patients with AKI, the associated mortality has declined.

## Key messages

• The incidence of AKI has increased over the past decade.

• AKI associated with ICU admissions for metabolic diagnoses and/or poisonings appears to have increased.

• AKI associated with ICU admissions for trauma has decreased.

• AKI carries an independent increased risk of death.

• The associated mortality for patients with AKI remains high but has declined over the past decade.

## Abbreviations

AKI = acute kidney injury; ANZICS = Australia New Zealand Intensive Care Society; APACHE = Acute Physiology and Chronic Health Evaluation; APD = Adult Patient Database; CI = confidence interval; ICU = intensive care unit; OR = odds ratio.

## Competing interests

The authors declare that they have no competing interests.

## Authors' contributions

SMB developed the study protocol, analysed data, and wrote and revised the manuscript. CG extracted the data from the ANZICS APD. RB conceived the study, assisted in developing the study protocol and provided critiques of successive drafts of the manuscript. All authors read and approved the final manuscript.
